# Pentagonal all-in-all-out antiferromagnetic chains in NaMn6Bi5

**DOI:** 10.1063/4.0000985

**Published:** 2025-10-27

**Authors:** Madalynn Marshall, Raimundas Sereika, Wenli Bi, Randy S. Fishman, David S. Parker, Huibo Cao

**Affiliations:** 1 Kennesaw State University, Kennesaw, Georgia, USA; 2 Department of Physics, University of Alabama, Birmingham, Alabama, USA; 3 Materials Science and Technology Division, Oak Ridge National Laboratory, Oak Ridge, Tennessee, USA; 4 Neutron Scattering Division, Oak Ridge National Laboratory, Oak Ridge, Tennessee, USA

## Abstract

Quasi-one-dimensional systems have garnered significant attention owing to the exotic properties they can host including superconductivity, charge density waves, topological spin excitations and more. Pressure-induced superconductivity has been realized in a new family of Mn-based Q1D materials, AMn6Bi5 (A = K, Rb, Cs, Na), with unique [Mn6Bi5]−1 double-walled columns. The smallest countercation Na+ yields the highest chemical pressure experienced in this family, reducing the Mn interatomic bond lengths and enhancing the metallicity and magnetic frustration within the Mn pentagonal antiprisms, thus, driving NaMn6Bi5 closer to the high-pressure superconducting phase. Distinct from the single magnetic transition in other family members, NaMn6Bi5 goes through multiple magnetic transitions at *T*N1 ∼88 K, *T*N2 ∼52 K and *T*N3 ∼48 K. In this talk I will present the findings of the unique low temperature, below *T*N3 ∼48 K, noncolinear “all-in-all-out” pentagonal antiferromagnetic order and high temperature in-plane moment dispersed pentagon phase in NaMn6Bi5 determined from single crystal neutron diffraction. The low temperature “all-in-all-out” state exhibits spins pointing all towards or away from the center of the pentagon and alternating down the Mn pentagonal antiprism columns along the *b* axis. The innermost central Mn-site continuously shows no/negligible ordered moment, resulting from the magnetic frustration within the Mn pentagonal antiprisms and nearly metallic bond distances. High pressure X-ray diffraction up to 18.5 GPa revealed no additional lattice transition, indicating the magnetic variation under pressure is highly relevant to the high-pressure superconducting phase found in this family. This investigation has, therefore, shed new light on the rare one-dimensional Mn-based superconductors.

